# Evaluation of a workplace intervention to promote commuter cycling: A RE-AIM analysis

**DOI:** 10.1186/1471-2458-13-587

**Published:** 2013-06-17

**Authors:** Veerle Dubuy, Katrien De Cocker, Ilse De Bourdeaudhuij, Lea Maes, Jan Seghers, Johan Lefevre, Kristine De Martelaer, Greet Cardon

**Affiliations:** 1 Department of Movement and Sport Sciences, Ghent University, Watersportlaan 2, Ghent B-9000, Belgium; 2 Department of Public Health, Ghent University, De Pintelaan 185, Ghent B-9000, Belgium; 3 Research Foundation Flanders, Egmontstraat 5, Brussels B-1000, Belgium; 4 Department of Kinesiology, KU Leuven, Tervuursevest 101, Leuven B-3001, Belgium; 5 Department of Movement and Sports Training, Vrije Universiteit Brussel, Pleinlaan 2, Brussels B-1050, Belgium

**Keywords:** Commuter cycling, RE-AIM, Workplace intervention, Physical activity

## Abstract

**Background:**

Originating from the interdisciplinary collaboration between public health and the transportation field a workplace intervention to promote commuter cycling, ‘Bike to Work: cyclists are rewarded’, was implemented. The intervention consisted of two cycling contests, an online loyalty program based on earning ‘cycling points’ and the dissemination of information through folders, newsletters, posters and a website. The study purpose was to evaluate the dissemination efforts of the program and to gain insights in whether free participation could persuade small and middle-sized companies to sign up.

**Methods:**

The RE-AIM framework was used to guide the evaluation. Two months after the start of the intervention a questionnaire was send to 4880 employees. At the end of the intervention each company contact person (n = 12) was interviewed to obtain information on adoption, implementation and maintenance.

Comparison analyses between employees aware and unaware of the program were conducted using independent-samples t-tests for quantitative data and chi-square tests for qualitative data. Difference in commuter cycling frequency was assessed using an ANOVA test. Non-parametric tests were used for the comparison analyses between the adopting and non-adopting companies.

**Results:**

In total seven of the twelve participating companies adopted the program and all adopting companies implemented all intervention components. No significant differences were found in the mean number of employees (*p* = 0.15) or in the type of business sector (*p* = 0.92) between adopting and non-adopting companies. Five out of seven companies had the intention to continue the program. At the individual level, a project awareness of 65% was found. Employees aware of the program had a significantly more positive attitude towards cycling and reported significantly more commuter cycling than those unaware of the program (both *p* < 0.001). Participation was mainly because of health and environmental considerations.

**Conclusions:**

The results of the dissemination study are promising. The adoption and implementation rates indicate that the ‘Bike to Work: cyclists are rewarded’ program seems to be a feasible workplace intervention. At the individual level, a higher score of commuter cycling was found among the employees aware of the program. Nevertheless, more evidence regarding long term effectiveness and sustainability of the intervention is needed.

## Background

The importance of regular physical activity (PA) in the maintenance of good health and the primary prevention of chronic diseases has been well documented [1-5]. Current public health recommendations for adults emphasize the need to accumulate 30 minutes of moderate to vigorous PA on most, preferably all, days of the week [6]. Within the field of public health, a variety of interventions to promote PA and to encourage people to meet this recommendation have been undertaken [7]. Health research has often focused on leisure-time or recreational PA [8]. However, PA interventions which relied upon recreational activities were found not to be very successful in increasing the levels of PA [9,10]. Possible explanations were reflecting inadequately the needs and interest of the target population and insufficiently addressing the barriers of participation (e.g. a lack of time) [9]. A different focus on the promotion of health enhancing PA emerged from the interdisciplinary collaboration between public health and transportation researchers [11,12]. Where the health promotion sector usually emphasizes leisure-time walking and cycling for health reasons, the transportation field were mainly interested in walking and cycling as elements of active commuting and sustainable mobility [13,14]. Focusing on cycling as a mode of transport used during trips to work may be a good strategy to increase PA in adults [15]. Commuter cycling provides an opportunity for health-enhancing PA because the built-in framework for regular practice enables commuters to engage in two daily activity bouts [16-18]. As it can easily be integrated into the daily routine [19] a more permanent adoption of the behavior is facilitated [20]. Furthermore, it offers attractive opportunities for a large number of people in the working population to reach the PA guidelines [18]. A maximum distance of 10 kilometers to the workplace was found to be a feasible commuter cycling distance [21]. So, for Flanders (Dutch-speaking northern part of Belgium with ±6 million inhabitants), this means that nearly half of the working population would be eligible for commuter cycling [22].

Besides the health-related benefits, commuter cycling is also beneficial to our environment and it contributes to the reduction of mobility problems [23-25]. Despite these various benefits, the 19% of the working population living within five kilometers of their work, cycle to work in Flanders, while the majority (53%) travels by car [26].

A Flemish cycling organization, promoting cycling as a key element of sustainable mobility [27], implemented a workplace intervention to promote commuter cycling, named ‘Bike to Work: cyclists are rewarded’. This intervention was based on effective evidence-based interventions to promote active transport in general and commuter cycling in particular [28,29]. Regarding the promotion of active transport, the Australian pilot study of a workplace project making use of posters, e-mail newsletters and an incentive to promote active transport, indicated a decrease of car trips in the weekend and a reduction of the proportion of participants driving to work [28]. Furthermore the review of Ogilvie et al. [29] showed a positive shift from cars to walking and cycling. Projects promoting commuter cycling by information materials endorsing the personal and environmental health benefits of cycling, providing bicycle facilities [30] and entering an element of competition resulted in an increase of commuter cycling and cycling in general [11,30,31].

The workplace setting was chosen as it allows reaching a substantial proportion of the adult population and it is believed to provide good opportunities to influence employee behavior [32-35]. After a successful implementation of the intervention in large companies the cycling organization also wanted to implement the intervention in small (< 100 employees) and middle-sized (< 800 employees) companies. The aim of the present study is to evaluate the dissemination efforts of the free workplace intervention ‘Bike to Work: cyclists are rewarded’ in small and middle-sized companies. Gaining insights in whether free participation can persuade small and middle-sized companies to participate in the program, was assessed as well.

The RE-AIM framework was used to guide the evaluation. This model has shown to be very useful for evaluating dissemination efforts and provides information on five intervention related factors: reach, effectiveness, adoption, implementation and maintenance [36,37].

## Methods

### Intervention implementation

The workplace intervention ‘Bike to Work: cyclists are rewarded’ was implemented in Flanders between May 2011 and March 2012 by the Flemish Cycling Union. This cyclist organization consists of volunteers and staff members who lobby for cycling policies at different policy levels. The intervention was implemented in twelve small and middle-sized companies (See Table 1) as an internet-based program and promoted commuter cycling to all employees. During the initial implementation a participation fee of the companies was asked. As it was found that small and middle-sized companies were put off by this participation fee, companies could now sign in for free. Similar to effective commuter cycling interventions in the Netherlands [31] and Germany [11,38] the intervention consisted of three major components: (1) two cycling contests, (2) an online loyalty program based on earning ‘cycling points’ and (3) the dissemination of information . To encourage potential cyclists to start cycling to work the first cycling contest took place during summer (months June-July 2011). To encourage employees to continue to cycle to work the second cycling contest took place during winter (October-November 2011). To participate, employees had to sign up as a team of at least five members. Each team was challenged to cycle half of the commuter trips by bike. Afterwards, of all teams that had met the challenge, three were randomly selected to receive a price (e.g. GPS, rain coat, backpack,…). The loyalty program aimed at motivating cyclists all year-round. Employees had to register on the ‘Bike to work: cyclists are rewarded’ website in order to track each cycling trip on their personal score page. Throughout the intervention the kilometers travelled by bike could be exchanged for gift vouchers, concert tickets, etc. Concurrently with the other intervention components, information was disseminated at the workplace through folders, newsletters, posters and a website. At the beginning of the program each company received an information package containing posters and folders to inform all employees of the program. Two newsletters were send to all employees to announce the start of the cycling contests and to encourage them to sign up. During the intervention period, the information on the website was available to all employees, in exception of the loyalty program where employees had to register.

**Table 1:** Overview participating companies

**Business sector**	**Number of companies**	**Number of employees *(subtotal)***
Service sector	3	330
		200
		490
		*(1020)*
Production	4	285
		400
		650
		235
		*(1570)*
Nursing	3	230
		120
		850
		*(1200)*
Education	1	650
		*(650)*
Distribution	1	640
		*(640)*
	**Total number of companies = 12**	**Total number of employees = 5080**

The selection of the companies was balanced across the five provinces of Flanders, as each provincial mobility center was asked to select three potential companies. The five mobility centers, one in each province, are funded by the Flemish government and provide advice and assistance on sustainable mobility to employers and employees. They were addressed as they have a clear view on the mobility policy of companies. The selection was based on the mobility profile of the company considering the number of employees living within a radius of 5 to 10 km to the workplace and employees having a regular work schedule. Furthermore the selection comprised companies from different types of business sectors and with different numbers of employees (see Table 1). Twelve of the fifteen contacted companies agreed to participate in the project. Each organization was asked to appoint a contact person responsible for the practical monitoring of the project. The Flemish Government provided funding for the implementation of the intervention, allowing companies to sign in for free.

### RE-AIM

The RE-AIM -model is an evaluative framework focusing on multiple criteria associated with health related behavior change research [36]. It helps to translate research findings into practice and it contributes to a balanced emphasis on both internal and external validity [39]. The framework conceptualizes the public health impact of an intervention as a function of five factors including reach, efficacy, adoption, implementation and maintenance [40]. The framework has previously been used to evaluate dissemination efforts for PA programs [41,42]. A short overview of the five dimensions is given below. For a full description please refer to Glasgow et al. [43].

Two dimensions operate at the individual level: reach and efficacy or effectiveness. *Reach* refers to the proportion and the characteristics of persons (i.e. patients or employees) who receive a program. *Efficacy* or *effectiveness*, depending on the study, concerns the impact of an intervention on important outcomes [36]. The dimensions ‘adoption’ and ‘implementation’ are both organizational-level measures. *Adoption* is the percentage and representativeness of settings that are willing to implement a health promotion program. *Implementation* deals with the extent to which various elements of a program are delivered as intended. The final dimension, *maintenance,* operates at both the individual and the organizational level. At the individual level, maintenance refers to the extent to which effects are stable long after an intervention is delivered. At the organizational level maintenance covers the extent to which a program becomes institutionalized or part of the routine practices of an organization.

### Data collection

Data were collected at the individual (i.e. employees) and the organizational (companies) level. To obtain data at the individual level the contact person of each company was asked to distribute an e-mail containing a link to a questionnaire among all employees. The first e-mail was sent during the month of June 2011, two months after the official start of the project (May 2011), followed by a reminder e-mail one month later. If respondents were unable to fill out the questionnaire online, a paper form was provided. The questionnaire was based on the survey of the Dutch intervention to promote commuter cycling [31] and consisted of three major parts: (1) demographic variables, (2) information on commuter cycling (e.g. commuter cycling frequency, attitude towards cycling, six months intention to commuter cycling, …), and (3) program components (awareness of the program, program appreciation,…).

Data at the organizational level were obtained at the end of the program by conducting a telephone-administered survey (based on the questions in Table 2) with the contact person of each company.

**Table 2:** Items used to assess the RE-AIM indicators

**Reach**			
**Awareness**	*Have you heard of the BtW*^*1 *^*program?*	□ Yes, I know the BtW program, but I am not registered on the online application	
		□ Yes, I know the BtW program and I am registered on the online application	
		□ No, I don’t know the BtW program	
**Motivators for participation**	*What motivated you to participate in the BtW program?*	□ I was planning to start cycling (or to cycle more) and this is program support me	□ Earning cycling point appealed to me
			□ A colleague convinced me
		□ I want to contribute to a better environment	□ I feel less satisfied with my car use
		□ It is good for my health	□ The idea of cycling together appealed to me
**Barriers to participation**	*What is the reason that you are not registered on the BtW program?*	□ It is not possible to cycle to work because of the long distance	□ Bad timing of the campaign
			□ I am not interested in cycling
		□ Cycling to work is difficult because of my work schedule	□ The campaign did not appeal to me
		□ Cycling to work is difficult because of my family situation	□ I already cycle to work and I don’t need a program to support me
			□ I don’t use a computer regularly
**Effectiveness**			
**Attitude towards cycling**	*How do you feel about cycling? (5-point scale: Fully agree – fully disagree)*	□ I am not the cycling type	□ Cycling is a healthy way of travelling
		□ Cycling offers freedom and flexibility	□ Cycling is dangerous
		□ Cycling is the fastest way of travelling	□ I find cycling stressful
**Commuter cycling frequency**	*Did you cycle to work in the past month?*	□ Yes, about less than once a week	□ Yes, about four times a week
		□ Yes, about once a week	□ Yes, about five times a week
		□ Yes, about twice a week	
		□ Yes, about three times a week	□ Yes, more than five times a week
			□ No, I did not cycle to work in the past month
**Adoption**			
**Program adoption**	*Did your company implement the BtW program*	□ Yes	
		No	
**Reason non-adoption**	*‘If your company did not implement the BtW program, what were the main reasons?*	Open ended	
**Implementation**			
**Company contact person**	*Which program components were carried out?*	□ *Dissemination of information*	
		□ *The online application*	
		□ *The organization of the cycling contests*	
**Employees**	*How would you assess the following components: (5-point scale: very positive – very negative)*	□ *The dissemination of information*	
		□ *The online application*	
		□ *The summer cycling contest*	
**Maintenance**			
**Organizational level**	*Does your organization have the intention to continue the BtW program next year?*	□ Yes	
		□ No	
		□ Maybe	
**Individual level**	*What are your intentions regarding commuter cycle in the future (next 6 months)*	□ *Planning to commuter cycle more often*	□ *Program did not change my behavior*
		□ *Planning less commuter cycling*	□ *Do not know yet*
		□ *Planning to stop commuter cycling*	
**Suggestions to promote commuter cycling**	*Do you have any suggestions on how your company could continue to promote commuter cycling?*	Open ended	

^1^BtW: Bike to Work: cyclists are rewarded.

The study protocol was approved by the Ethics Committee of the Ghent University.

### RE-AIM evaluation

An overview of the items from the questionnaire and the telephone-administered interview used to assess the RE-AIM indicators is presented in Table 2.

### Reach

Project awareness was assessed using the question *‘Have you heard of the program?’*. Those answering ‘*No, I don’t know the program’* were considered unaware of the program, those answering one of the two other options (*Yes, I know the ‘Bike to Work: cyclists are rewarded’ program, but I am not registered on the online* application and ‘*Yes, I know the ‘Bike to Work: cyclists are rewarded’ program and I am registered on the online application’*) were considered aware of the program. Representativeness was estimated by comparing differences in age, gender, body mass index (BMI) and perceived health between those aware versus those not aware of the program.

Employees aware of the program and being registered, were asked about the main motivators for participation. Employees that indicate knowing the intervention but who did not register were asked about the main barriers to participation.

### Effectiveness

The intervention was based on effective interventions to promote commuter cycling. Therefore the effectiveness of the evidence-based program was assessed, in line with the study of Van Acker et al. [41] by comparing the general attitude towards cycling and the frequency of commuter cycling between employees aware of the program and those unaware of the program.

### Adoption

To establish adoption of the program, all contact persons were asked if their company implemented the program. The main reason for non-adopting was asked if the program was not implemented. Representativeness of the settings was assessed by comparing the type of business sector and the number of employees between the adopting and non-adopting companies.

### Implementation

To gain insights in the implementation of the program, each contact person was asked about the different program components that were carried out. Furthermore, employees were asked to evaluate the different program components.

### Maintenance

Maintenance was assessed at both the individual as the organizational level. At organizational level, the companies were asked about their intention to continue the program. Employees aware and registered to the program were asked about their intention to commuter cycle during the next six months to establish maintenance at individual level. Employees aware and unaware of the program were asked for suggestions on how their company could continue to promote commuter cycling.

### Data analysis

Independent samples t-tests and chi square tests were used to compare demographic characteristics (age, gender, BMI and perceived health) between employees aware and unaware of the program. As the travel distance to work was skewed, comparison analyses between employees aware and unaware of the program were conducted by using an independent samples median test. Difference in the attitude towards cycling and the frequency of commuter cycling among employees who were aware of the program and those who were not aware of the program was assessed using an ANOVA test. To establish representativeness of the companies, comparison analyses between the adopting and non-adopting companies were conducted using a Mann- Whitney *U* test for quantitative data (number of employees) and a chi-square test for qualitative data (type of business sector). All data were analyzed using SPSS 20.0 for Windows and an α level of *p* < 0.05 was used to decide upon statistical significance.

## Results

All companies, except one, distributed the questionnaire to all employees (n = 4880). After two mailings a total of 1116 respondents (23%) completed the questionnaire. All contact persons of the twelve companies agreed to participate in the telephone-administered interview.

**Figure 1:**
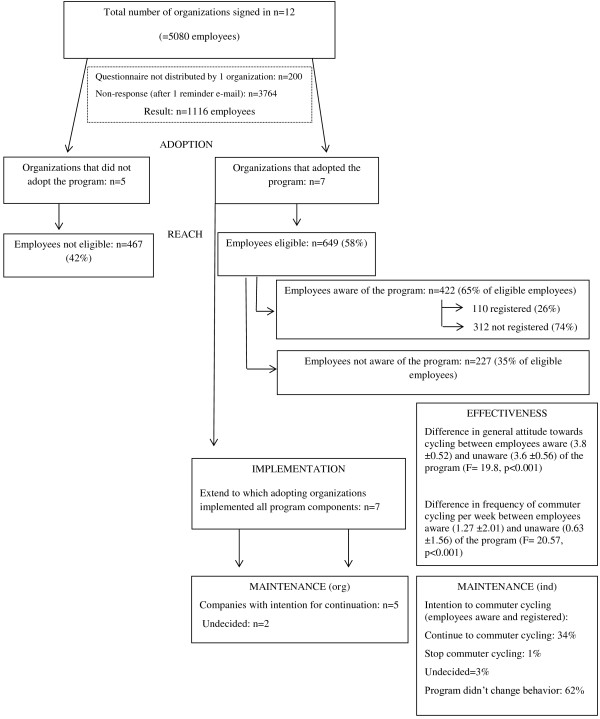
Overview of the different dimensions of the RE-AIM framework applied to the 'Bike to Work: cyclists are rewarded' program.

The main results for all the RE-AIM dimensions are presented in Figure 1.

### Adoption

#### Organizational level

Seven of the twelve companies adopted the intervention. The remaining five companies who did not adopt the ‘Bike to Work: cyclists are rewarded’ program were asked about the reason for not participating. The main reported reasons were a lack of time and a shortage of staff resulting in a lack of monitoring and managing of the program. To establish representativeness, comparative analyses between adopting and non-adopting companies were conducted. No significant differences were found in the mean number of employees (*p* = 0.15) or in the type of business sector between adopting and non-adopting companies (*p* = 0.92).

### Implementation

#### Organizational level

All adopting companies (n = 7) reported having implemented all ‘Bike to Work: cyclists are rewarded’ program components (dissemination of information, the online application and the organization of the two cycling contests). Additionally, employees who were aware and registered on the program were asked to evaluate the different program components (n = 110).

#### Individual level

In general the dissemination of information was evaluated relatively positively. More than half of the employees was positive about the website and the folder. Meanwhile a substantial proportion (15%) of the employees reported not knowing the newsletter. The contest as well as the online application where cycling points could be exchanged for gift vouchers and coupons were evaluated positively by slightly more than one third of the employees. No strong opinion on the contest and online application was noted as about half of the employees remained indecisive (See Table 3).

**Table 3:** Evaluation of intervention components

	**Positive**	**Negative**	**Undecided**	**Unknown**
**Dissemination of information**				
Website	53%	3%	38%	6%
Folder (cycling calendar)	59%	1%	33%	7%
newsletter	27%	0%	58%	15%
**Contest**	35%	4%	53%	8%
**Online application**	33%	3%	52%	12%

### Reach

#### Individual level

Of all 1116 employees who filled out the questionnaire, 649 received the intervention (employees of the adopting companies) and were thereby eligible (58%). Of all 649 eligible employees, 65% was aware of the ‘Bike to Work: cyclists are rewarded’ program (n = 422). Of those employees aware of the program, 26% (n = 110) was registered on the online application. The median travel distance to work was comparable for employees aware (12.5 km) and unaware (13.5 km) of the program (*p* = 0.7). The median travel distance to work differed between employees being aware of the program and being registered (8 km) and employees being aware but not being registered (13 km; *p* = 0.07).

The main reported motivators to register and participate in the ‘Bike to Work: cyclists are rewarded’ program were ‘It is good for my health’ (100% agreed) and ‘I want to contribute to a better environment’ (97% agreed). The least of the employees agreed with the statements ‘The idea of cycling together appealed to me’ and ‘A colleague convinced me’ as motivators for participation (See Table 4). Employees that did not register to the online application but were aware of the program were asked about the barriers to participate in the ‘Bike to Work: cyclists are rewarded’ program. The most frequently reported reasons were: the distance to work (20%), the family situation (9%), already cycling to work and having no need for a program to support this (7%) and the work schedule (7%). Only 2% mentioned that the campaign did not appeal to them and 2% indicated that cycling does not interest them.

**Table 4:** **Main reported motivators to participation** (**employees being aware and registered)**

**Motivators to register and participate in the ‘Bike to Work: cyclists are rewarded’ program**	**% agreement**
It is good for my health	100%
I want to contribute to a better environment	97%
I feel less satisfied with my car use	56%
Earning cycling points appealed to me	51%
I was planning to cycle more and the program supports me	43%
The idea of cycling together appealed to me	33%
A colleague convinced me	31%

Results regarding the representativeness revealed that those aware of the project were significantly older (39.36 years, SD 10.7) than those unaware (35.19 years, SD 9.2; *p* < 0.001) of the program, but groups did not differ in gender, BMI or perceived health. Therefore further analyses on attitude towards cycling and commuter cycling frequency were controlled for age.

### Effectiveness

#### Individual level

Results showed that the general attitude towards cycling was significantly (*p* < 0.001) more positive among employees aware of the project than those unaware (respectively mean positive attitude of 3.8 ±0.52 and 3.6 ±0.56). Analyses revealed that employees aware of the project – next to having a more positive attitude towards cycling – also reported significantly more commuter cycling per week than those not aware (respectively 1.27 ± 2.01, 0.63 ±1.56, *p* < 0.001).

### Maintenance

#### Organizational level

Results concerning maintenance at the organizational level revealed that five of the seven adopting companies had the intention to continue the ‘Bike to Work: cyclists are rewarded’ program next year, the two remaining companies were undecided.

#### Individual level

At the individual level, employees were asked about their intentions to cycle within the next six months. About 34% of the employees was planning to continue commuter cycling, only 1% was planning to stop commuter cycling, and 3% did not know it yet. In total, 62% indicated that the program did not change their commuter cycling behavior.

All employees were asked for suggestions on how the company could promote commuter cycling. The main suggestions – made by both aware and unaware employees concerned cycling accommodation (i.e. a covered and safe bicycle shed, bike racks, rainwear, locker room, shower facilities) and a higher bicycle compensation. Furthermore, employees being aware and registered on the program also reported that participation in commuter cycling projects should be promoted more actively and accompanied by regular communication. In addition flexible working hours and a bicycle repair service were two suggestions made by employees being aware but not registered on the program.

## Discussion

Within the field of health promotion several effective workplace interventions to promote PA in general have been developed [44], however few studies report on the dissemination and implementation of these interventions [37,45]. Nevertheless, a thorough evaluation of these workplace interventions is necessary as it can provide valuable information on the level of intervention implementation and fidelity, feasibility and effectiveness of an intervention, and it is needed to identify ways to improve practice [36].

The current study used the RE-AIM framework to assess individual and organizational factors associated with a workplace intervention to promote commuter cycling. In general, the intervention ‘Bike to Work: cyclists are rewarded’ was well received by the different companies and employees. Similar to the Dutch intervention to promote commuter cycling the online application, the website and folders were assessed positively. Also, the main reported reasons for participation i.e. health and environmental concerns, were confirmed in the present study [31]. The finding that half of the employees was motivated by the possibility to earn ‘cycling points’ was somewhat in contrast to the Dutch study where only 14% indicated this. The main reported barriers to participation were not linked to the intervention or the content of the intervention, but rather practical in nature (e.g. distance to work). This is in contrast to the Dutch study where almost 30% indicated being insufficiently familiar with the campagne and only 5% stated that distance to work was a barrier to participation [31]. A possible explanation is that the current study had a higher proportion of participants living further away from the workplace. It was noted that, although it was only intended to select companies easily accessible by bike (within a radius of 5 to 10 km to the workplace), the median distance to work for employees aware but not registered and employees unaware of the program was 13 kilometer.

The positive results on the attitude towards cycling and the difference in commuter cycling frequency are a conservative indication of the intervention effectiveness. The results on commuter cycling are in line with other cycling interventions [29-31]. Noteworthy is that, despite the increase in commuter cycling, more than half of the employees stated that the program did not change their behavior. A possible explanation could be that these observed increases in cycling are largely to be attributed to existing cyclists making more cycling trips rather than to ‘new cyclists’ [29].

Limitations of the current results are noted. Firstly, this study evaluated a workplace intervention to promote commuter cycling within a real world setting, which is both a strength as a limitation. The absence of a non-intervention group of companies is a methodological weakness, so employees results concerning effectiveness need to be interpreted with caution. Also, the cross-sectional nature of the data makes it difficult to adequately address the maintenance dimension. Another limitation is the low response rate of the employees. Although a reminder e-mail was sent, a high percentage of non-response remained, resulting in a negative impact on the representativeness of the sample. At the same time the lack of information on non-participating employees is a weakness of the present study. Furthermore, self-reported data can lead to social desirable answers. For respondents aware of the program, questions concerning cycling frequency may have been answered in line with the social tendency. In line with the abovementioned limitation, the implementation of the program was not assessed objectively. The contact person of each organization was interviewed by telephone and asked to which degree all program components were carried out. Combining this telephone-administered survey with an ‘on site’ observation could contribute to a more comprehensive view of the program implementation.

The use of the RE-AIM model to guide the evaluation is a strength, as it ensures a more comprehensive evaluation of the intervention. Next to the effectiveness of the program, insights were also gained into the reach, adoption and maintenance of the intervention. This is valuable information considering that the benefit of any public health intervention is not only determined by its efficacy or effectiveness, but also by the extent to which it is appropriately adopted and implemented [45]. The evaluation revealed that both health and environmental considerations seem to be good arguments to encourage employees to start commuter cycling. The finding that the provision of adequate and safe cycling accommodation by the company would be an additional stimulant for employees confirms the findings of several other studies where safe bicycle parking, bike enclosures and bike racks were considered to be important by cyclist [46-49]. In addition, insights were gained in the role of cycling distance to work as a barrier to participation. It seems that journeys over 10 km discourage employees to cycle to work. This finding is in line with the study of Iacono et al. [21] reporting that the majority of the trips to work fall within a 10 km distance.

Furthermore this study contributes to the limited body of dissemination literature [50]. Although the need for more dissemination studies has already been emphasized [51], this study is – to our knowledge – the first dissemination study on a workplace intervention to promote commuter cycling.

Future dissemination of the intervention needs to take into consideration the main reported barrier to adoption, being a lack of time to monitor the program. The practical implementation of the intervention should not be additional to one’s current job responsibilities, but extra time for monitoring of the program should be provided. Furthermore for small and middle-sized companies, it seems that providing these companies the opportunity of initially signing in for free does not encourage future paid participation. Also, companies need to consider the main barriers to participation, being the distance to work and the work schedule of the employee, when deciding to implement the intervention.

## Conclusions

In conclusion, the results of this dissemination study are promising. The adoption and implementation rates indicate that the ‘Bike to Work: cyclist are rewarded’ program seems to be a feasible workplace intervention. At the individual level, a higher score of commuter cycling was found among the employees aware of the program In addition, the employees gave a positive evaluation of the intervention. Nevertheless more evidence regarding long term effectiveness and sustainability of the intervention is needed.

## Abbreviations

PA: Physical activity; BMI: Body mass index.

## Competing interests

The authors declare they have no competing interests.

## Authors’ contributions

VD contributed to the design of the study (in consultation with De Fietsersbond), analyzed the data, led the writing of the paper and wrote the manuscript. IDB, LM, GC and KDC participated in the design of the study, helped to interpret the data and to draft the manuscript. KDM, JS and JL provided feedback on the manuscript. All authors read and approved the final manuscript.

## Pre-publication history

The pre-publication history for this paper can be accessed here:

http://www.biomedcentral.com/1471-2458/13/587/prepub
